# The Correlation of Computerized Scoring in Home Sleep Apnea Tests with Technician Visual Scoring for Assessing the Severity of Obstructive Sleep Apnea

**DOI:** 10.3390/jcm13144204

**Published:** 2024-07-18

**Authors:** Colton Hawco, Amrita Bonthu, Tristan Pasek, Kaylee Sarna, Laurence Smolley, Anas Hadeh

**Affiliations:** 1 Cleveland Clinic Florida, Weston, FL 33331, USA; 2 School of Medicine, Case Western Reserve University, Cleveland, OH 44106, USA

**Keywords:** home sleep apnea test, obstructive sleep apnea, Polysmith, artificial intelligence, automated scoring, computer-assisted diagnosis, diagnostic accuracy

## Abstract

**Background**: Obstructive sleep apnea (OSA) affects a significant proportion of the global population, with many having moderate or severe forms of the disease. Home Sleep Apnea Testing (HSAT) has become the most common method of diagnosing OSA, replacing in-lab polysomnography. Polysmith software Version 11 by Nihon Kohden allows for the automatic scoring of respiratory events. This study aimed to assess the validity of this technology. **Study Objectives:** The objective was to assess the accuracy of the Polysmith Software Automatic Scoring Algorithm of HSATs in comparison to that of sleep technicians. **Methods:** One hundred twenty HSATs were scored by both sleep technicians and Polysmith software. The measured values were the respiratory event index (REI), apneic events, and hypopneic events. Agreement between the two methods was reached by utilizing the Kruskal–Wallis test, Pearson correlation coefficient, and Bland–Altman plot, as well as sensitivity, specificity, positive predictive value (PPV), and negative predictive value (NPV). **Results:** The correlation between the REI calculated by the software and technicians proved to be strong overall (r = 0.96, *p* < 0.0001). The mild OSA group had a moderate correlation (r = 0.45, *p* = 0.0129). The primary snoring, moderate OSA, and severe OSA groups showed stronger correlations (r = 0.69, *p* < 0.0001; r = 0.56, *p* = 0.012; r = 0.71, *p* < 0.0001). The analysis conducted across all groups demonstrated an average sensitivity of 81%, specificity of 94%, PPV of 82%, and NPV of 94%, with an overall accuracy of 81%. When combining the moderate and severe OSA groups into a single category, the sensitivity was 90%, specificity was 100%, PPV was 100%, and NPV was 91%. **Conclusions:** OSA can be reliably diagnosed from HSATs with the automated Polysmith software across all OSA disease severity groups, with higher levels of accuracy in moderate/severe OSA and lower levels of accuracy in mild OSA.

## 1. Introduction

Obstructive sleep apnea (OSA) affects around 22% of the global population, with many having moderate or severe forms of the disease [1]. OSA is a sleep-related breathing disorder that is characterized by repetitive episodes of complete or partial upper airway obstruction during sleep [2]. These obstructions can manifest as primary snoring (asymptomatic non-apneic snoring [3]), hypopnea (reduction in airflow), and apnea (cessation of airflow) [4]. Consequently, untreated sleep apnea heightens the risk of various health complications, including hypertension, arrhythmias, pulmonary hypertension, and right and left ventricular failure, and it potentially increases mortality [5,6].

Recognizing the disease early and providing suitable treatment is crucial. Early detection and appropriate therapy can improve neurobehavioral outcomes and cardiovascular health [7]. With prompt identification of the pathology, continuous positive airway pressure (CPAP) or other therapies such as mandibular advancement devices may be provided to ameliorate the condition [6]. Early detection is limited by a shortage of sleep technicians, along with the high costs of conducting sleep studies [8,9,10]. When compared with in-lab polysomnography, HSATs have proven to be an effective means of diagnosis for OSA, in addition to being markedly less expensive [11,12].

Although HSATs are less resource-intensive than in-lab sleep studies, manual scoring remains a costly, challenging, and resource-consuming process [13]. Based on the current literature regarding polysomnographic analysis, artificial intelligence (AI) has shown promise in accurately providing a diagnosis of OSA while also saving time and mitigating the potential for human error [14,15,16]. Emerging data indicate that AI can effectively quantify the risk of OSA severity based on patient clinical features and subjective questionnaires [17]. However, there is still a significant gap in the implementation of a robust tool for providing an official diagnosis of OSA by way of automated scoring technology for HSAT data, especially because most of the current literature examining the use of AI for the diagnosis of sleep disorders has focused on the use of this technology for in-lab polysomnography or attended sleep studies data [9]. One software program currently available for the analysis of HSAT data is the sleep diagnostic software offered by Nihon Kohden (based in Tokyo, Japan): Polysmith Version 11. In prior studies, Polysmith Version 11 has proven its ability to be utilized in the analysis of sleep stage scoring [18].

The Polysmith Version 11 manual describes a validation study involving 7606 30 s epochs scored by both the Polysmith software and two registered sleep clinicians. The study reports high concordance rates for automated and manual scoring, with a 97.28% agreement for apnea events (CI: 1.32%) and 95.44% agreement for hypopnea events (CI: 2.55%) [19]. Despite these findings, this validation was based on a limited sample and lacked subgroup analyses. Our research aims to address these limitations by expanding the sample size and incorporating subgroup analysis to align with clinical practices where treatment strategies for sleep apnea vary by severity. This subgroup stratification is essential, as moderate-to-severe cases are associated with increased risks of neurological and cardiovascular complications; this stratification is supported by the practice parameters set by the American Academy of Sleep Medicine (AASM) [20], which recommends tailoring treatments based on the severity of sleep apnea to improve patient outcomes.

Given the prevalence of OSA, it is imperative to provide an expedient diagnosis with minimal cost and strain on labor resources. In this study, we aimed to evaluate the effectiveness of the Polysmith Version 11 sleep diagnostic software to achieve such a task.

## 2. Methods

A comparison between the scoring methods of Polysmith Version 11 AI software and technician visual scoring (gold standard) to interpret apneas, hypopneas, and the respiratory event index (REI) was conducted. Patient data were de-identified and aggregated. This study was deemed Exempt Human Subjects Research by the Institutional Review Board IRB# FLA 23-011. Data regarding clinical, demographic, and diagnostic results were recorded in an encrypted database. A power calculation assumed that the Pearson correlation coefficient (r) in the REI between the Polysmith Version 11 software and the technician would be greater than 0.80, and it expected an observed r of 0.85. A sample size of 100 would achieve an 80% power score, with a significance level of 5% for testing the hypothesis (H_0_: r ≤ 0.80 vs. H_1_: r > 0.80).

To determine the diagnostic categories of the patients, scoring was performed by two registered polysomnographic technologists (RPSGTs) with more than five years of experience who utilized the AASM scoring manual, version 2.6. Visual scoring was performed completely independent of assistance from the computerized algorithm. The RPSGTs involved in scoring participated monthly in the inter-scorer reliability assessment program administered by the American Academy of Sleep Medicine. This program provided them feedback by reviewing the Gold Standard comparison to see how their results measured against the experts in the field. The HSAT device used by the patients was the Nomad [21] portable device manufactured by Nihon Kohden, which records data for respiratory effort, nasal airflow/snoring, oxygen saturation (SpO_2_), and heart rate.

### 2.1. Selection Criteria and Diagnostic Categories

Data were collected from sleep studies performed from the first half of 2023 at Cleveland Clinic Florida’s Sleep Disorder Center. Patients (age 18 to 85) were selected based on a high suspicion of having sleep apnea, determined through clinical evaluation and questionnaires. Exclusion criteria included significant comorbidities such as heart failure, COPD, interstitial lung disease, and obesity hypoventilation syndrome. In addition, patients with comorbid sleep diagnoses such as insomnia, parasomnia, and narcolepsy were also excluded. This study included 120 patients divided into four equal groups of 30 individuals each: those with snoring, mild OSA, moderate OSA, and severe OSA. Consecutive sleep studies were assessed, and the first 30 individuals diagnosed with each condition were sorted into respective diagnostic groups.

### 2.2. Statistical Analysis

To gauge the correlation of analyses, particularly the REI between software and technician scoring, we used the Pearson correlation coefficient. This approach facilitated the development of an integrative model to assess the concordance between sleep technician and software scoring. For the evaluation of hypopneic and apneic events identified by both software and sleep technicians, the Kruskal–Wallis test was preferred over ANOVA due to the non-normal distribution of the data. To quantify the magnitude of the difference in the REI between the software and technician assessments, a *t*-test was applied.

Agreement was further scrutinized through chi-square tests, coefficient analyses, Bland–Altman plots, and tests of validity such as sensitivity, specificity, positive predictive value (PPV), and negative predictive value (NPV), collectively offering a comprehensive overview of the consistency between the two evaluation methodologies. The entire analysis was executed using SAS, version 9.4.

### 2.3. Polysmith Version 11 Scoring

Data procured from HSATs were provided to Polysmith Version 11 (proprietarily owned by Nihon Kohden) for interpretation. The software analyzed the incidence of hypopneic events as well as apneic events, which was ultimately compiled to produce the REI, defined as total apneas and hypopneas divided by the monitoring time. Both classifications of hypopneas were utilized, oxygen desaturation of ≥3% (AASM Criteria) or of ≥4% (Center for Medicare and Medicaid Services criteria). Hypopneas were aggregated using both criteria, with criteria selection depending on the patient’s insurance coverage requirements. Patients were grouped into 4 categories: snoring (no OSA) (REI: 0–4), mild OSA (REI: 5–14.9), moderate OSA (REI: 15–29.9), and severe OSA (REI >30). Other data extracted from the HSATs included the subjects’ nadir oxygen saturation, time below 89% oxygen saturation, and average heart rates.

## 3. Results

We analyzed 120 patients across the four severity groups (primary snoring, mild OSA, moderate OSA, and severe OSA) with 30 patients in each group. The mean age of the sample was 55.7 years of age, with 66 males and 54 females (Table 1). The mean recording time for all subjects was 440.2 min with an SD = 95.4. The correlation between the REI calculated by the software and technicians proved very strong overall (r = 0.96, *p* < 0.0001) (Table 2). The mild OSA group had a moderate correlation (r = 0.45, *p* = 0.0129), while the primary snoring, moderate, and severe OSA groups showed strong correlations within their respective groups (r = 0.69, *p* < 0.0001; r = 0.56, *p* = 0.012; r = 0.71, *p* < 0.0001). Hypopneic incidents were reported at a higher rate by the software when compared to technician scores (median difference of 19, IQR = 45, *p* < 0.0001), while apneic episodes were reported at a lower rate (median difference of −16.5, IQR 37.5, *p* < 0.0001). The cumulative median difference in REI between the software and technicians was 0.5 (IQR of 4.1); while statistically significant, this is clinically insignificant. An evaluation across all groups revealed an average sensitivity of 81%, specificity of 94%, PPV of 82%, and NPV of 94%, with an overall accuracy of 81%. When the moderate and severe OSA groups were merged, the sensitivity rose to 90%, the specificity reached 100%, the PPV was 100%, and the NPV was 91%.

### 3.1. Patient Characteristics Classified by Disease Severity

The patient characteristics are described above (Table 1).

### 3.2. Comparison of the Interpretation of Sleep Events by Software vs. Technicians

Regarding the difference in the number of hypopneas determined by software vs. sleep technician scoring (Table 3), the technician was found to identify a median of 19 more hypopneic events per study than the software across all groups, with a *p* < 0.0001. This trend persisted within each individual severity group, with technicians consistently scoring a higher amount of hypopneic events compared to software. Regarding apneic events, sleep technician analysis demonstrated a median of 16.5 fewer apneic events across all groups (*p* < 0.0001). This trend was also persistent across each individual severity group.

### 3.3. Software Diagnoses vs. Technician Diagnoses

**Figure 1:**
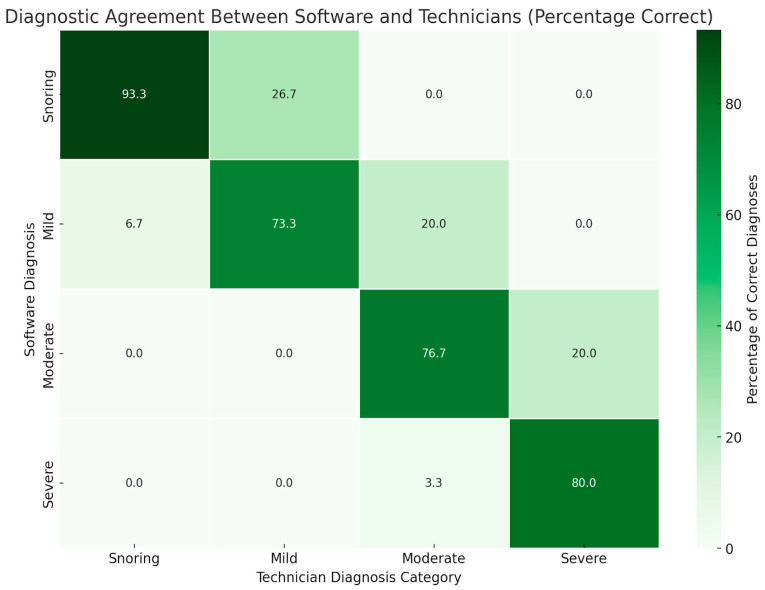
Heatmap of diagnostic concordance between the technicians and software across OSA severities. The color indicates the degree of agreement in the diagnostic category, with a deeper shade of green corresponding to higher agreement.

The agreement between the final diagnosis (shown in Table 4) provided by both technicians and software was evaluated. Among the 30 subjects previously diagnosed by technicians with mild OSA, 22 retained the diagnosis of mild OSA, while 8 were reclassified as snoring. For the 30 subjects initially diagnosed with moderate OSA, 6 were downgraded to mild OSA, 23 maintained the diagnosis of moderate OSA, and 1 was newly classified as severe OSA. Among the 30 subjects with a prior technician diagnosis of severe OSA, 24 were reaffirmed as severe OSA, and 6 were now categorized as having moderate OSA. Lastly, of the 30 subjects initially diagnosed as primary snoring by technicians, 2 were reclassified as mild OSA, while 28 retained the diagnosis of primary snoring. The heatmap (Figure 1) visually demonstrates this pattern of diagnostic agreement.

### 3.4. Correlation of REI between the Technicians and Software

**Figure 2:**
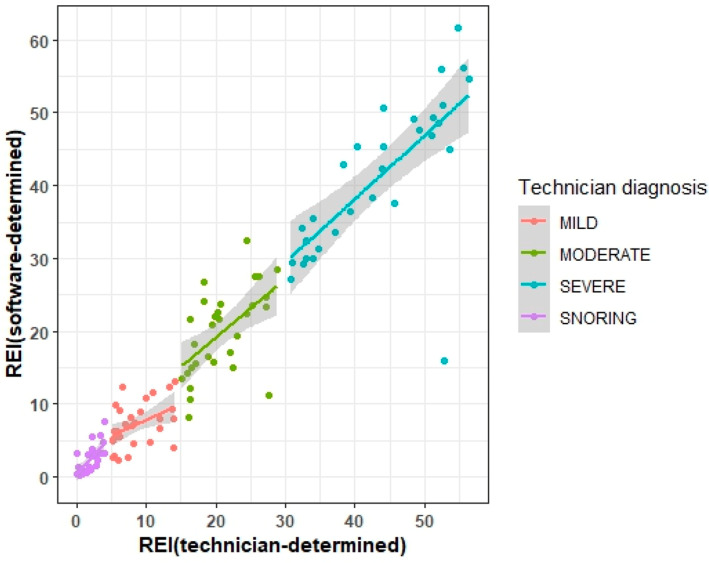
Visual demonstration of the degree of REI agreement between the technicians (line) and the software (points) by diagnostic category.

The REI across the entire study population displayed a statistically significant, strong correlation, with r = 0.96 and *p* < 0.0001. In the mild OSA group, both technician and software analyses exhibited a moderate correlation, with r = 0.45 and *p* = 0.0129, and this correlation was statistically significant. Across the snoring, moderate OSA, and severe OSA groups, the REI derived from software analysis did not differ significantly from technician evaluation, demonstrating a strong correlation between the analyses with r = 0.69 (*p* < 0.0001), r = 0.56 (*p* = 0.0012), and r = 0.71 (*p* < 0.0001), respectively (Table 5). This is visually demonstrated in Figure 2.

**Figure 3:**
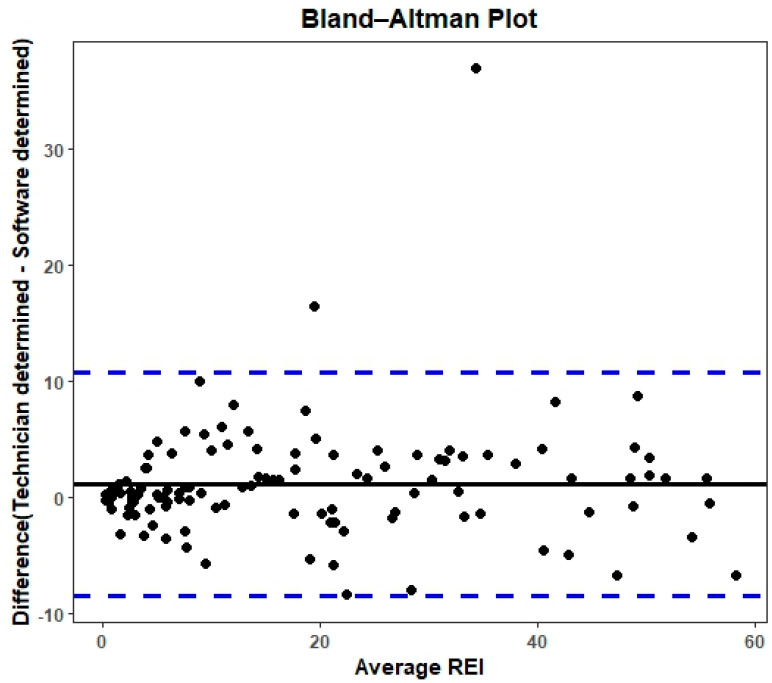
Bland–Altman plot. Upper and lower blue dashed lines indicate +1.96SD = 10.7 and −1.96 = −8.6, respectively. Solid horizontal black line indicates mean = 1.1.

The Bland–Altman plot (Figure 3) reveals a clustering of most points above and below the zero-difference line within the limits of agreement. This pattern indicates significant agreement between the two measurement methods without evidence of excessive systematic bias.

### 3.5. Sensitivity, Specificity, PPV, and NPV

Sensitivity, specificity, PPV, NPV, and accuracy were calculated for OSA groups. The snoring OSA group had the highest sensitivity at 93%, 91% specificity, 78% PPV, and 98% NPV. The mild and moderate OSA groups had the lowest percentages. The mild OSA group had 73% sensitivity, 91% specificity, 73% PPV, and 91% NPV. The moderate OSA group had 77% sensitivity, 93% specificity, 79% PPV, and 92% NPV. The severe OSA group had the second greatest percentages with 80% sensitivity, 99% specificity, 96% PPV, and 94% NPV. The average across all groups was 81% sensitivity, 94% specificity, 82% PPV, and 94% NPV. Accuracy across the entire population was 81%. Moderate/severe categories were combined to evaluate whether that would influence validity. The values for the moderate/severe group were notably high. When the moderate and severe OSA groups were combined into one category, a sensitivity of 90%, specificity of 100%, PPV of 100%, and NPV of 91% were calculated (Table 6).

## 4. Discussion

The aim of our study was to compare the effectiveness of the computerized scoring of respiratory events with the gold standard; visual scoring was performed by experienced RPSGTs. The Polysmith Version 11 automatic scoring algorithm proved to be a robust tool for use in analyzing HSATs across a spectrum of disease severity. In those with no sleep apnea (primary snoring), moderate OSA, and severe OSA, the results derived from software analysis were remarkably similar to that of the sleep technicians.

The difference in REI across all subjects was found to be a median of 0.5, with an interquartile range of 4.1, so it is unlikely that the ultimate diagnosis of OSA severity would be significantly impacted by using AI interpretation. Despite differences in classifying apneic and hypopneic events between software and technicians, the REI showed a strong correlation regardless because REI is based on the total number of sleep events, not their specific classifications. As the magnitude of differences observed in hypopneas and apneas reported is comparable between technician and software assessments, the final calculated REIs were similar.

Moreover, the Polysmith Version 11 software had high sensitivity, specificity, PPV, and NPV scores across diagnostic categories overall. Of note, the moderate/severe OSA group were very accurately detected by the automatic scoring system with a sensitivity of 90%, specificity of 100%, PPV of 100%, and NPV of 91%. This is a particularly important group to identify because they require more aggressive therapy such as CPAP. Patients with less severe sleep-related breathing disorders, snoring, or mild OSA are more likely to only require lifestyle changes such as diet, exercise and/or medication for weight loss, cessation of smoking, and/or avoidance of alcohol or sedative drugs near bedtime, as recommended by their clinical care givers.

The lowest sensitivity was for the mild OSA group (73%), indicating that the software may miss or underdiagnose milder cases. Such a conclusion is supported by the comparatively low correlation in the mild OSA group; r = 0.45 (*p* = 0.0197). It is noteworthy that the relatively low total number of sleep events associated with mild OSA might amplify the impact of even minor discrepancies in reported sleep event counts, potentially influencing this study’s findings. In addition, the software tended to score more apneas and less hypopneas than the technicians (Table 3); the detection algorithm may be more sensitive to apnea. This limitation suggests that Polysmith Version 11 is more reliable for confirming the presence of more severe OSA and may require supplementary diagnostic methods such as a review by RPSGTs or board-certified sleep specialists for the early detection of less severe cases.

Our study is subject to several limitations that we aim to address in future research. One notable limitation is the lack of extraction and analysis of comprehensive health data. Consequently, the presence of undiagnosed comorbid conditions might contribute to outliers in the data. Conditions such as obesity hypoventilation syndrome, comorbid lung disease, or cardiovascular issues could potentially compromise the accuracy of HSAT results and elevate the risk of obtaining unrepresentative outcomes [22,23,24]. Recognizing these limitations, future studies will incorporate a more thorough examination of individual health profiles and comorbidities to enhance the robustness and applicability of our findings. It is essential to acknowledge the potential influence of human error on the data, which could potentially underestimate the accuracy of Polysmith Version 11. Although sleep technician interpretations of HSAT data are typically deemed the gold standard, the inherent risk of inaccuracies in interpreting sleep events and diagnoses due to human error cannot be dismissed [25]. To mitigate this concern, future studies should incorporate inter-rater reliability testing, involving the averaging of scores from multiple technicians. This approach could help alleviate the impact of individual human errors and provide a more robust assessment of the technology’s accuracy. Further sources of potential inaccuracies are rooted in the technical malfunctions that HSAT hardware may encounter. These malfunctions, such as issues with the thermistor [26], detachment of sensors [27], and defects or missing data related to oximeter contact [28], could significantly impact the accuracy of acquired data.

Considering the future integration of AI into clinical practice, upcoming studies will focus on identifying the most suitable patients for HSAT evaluation by AI. Numerous studies have highlighted significant performance variability when comparing sleep technician analysis of HSATs to polysomnography, with potential links to pre-testing the suspicion of OSA within a population [29]. A prospective approach for future deployment involves employing AI scoring for all patients, supplemented by a second round of human sleep technician scoring specifically for individuals identified as having mild OSA by AI software. This combined strategy aims to optimize patient selection, leveraging the strengths of AI while ensuring a thorough evaluation for enhanced clinical accuracy.

With complications of untreated OSA [30] imposing an estimated financial burden of over 140 billion USD, a faster diagnosis is critical to address this economic strain [31]. Moreover, by reducing the labor required for HSAT analysis, AI can streamline patient care and enhance efficiency. This advancement will play a vital role in mitigating health disparities in sleep medicine, especially given the current shortage of sleep technicians and resources.

## 5. Conclusions

The Polysmith Version 11 software by Nihon Kohden has showcased its ability to interpret HSAT data at a level comparable to that of a sleep technician. Across various severities of OSA, the software consistently and accurately analyzes sleep data and events. The software especially excels in diagnosing moderate and severe cases. However, for cases diagnosed as mild OSA, further review by a technician or certified sleep physician is recommended. This promising performance suggests that, upon widespread integration into clinical practice for the analysis of HSATs, AI has the potential to significantly advance the field of sleep medicine.

## Figures and Tables

**Table 1:** Characteristics of OSA patients grouped by diagnostic category.

Variable	Total = 120	Snoring (*n* = 30)	Mild (*n* = 30)	Moderate (*n* = 30)	Severe (*n* = 30)	*p*-Value
Age, mean (SD)	55.7 (13.3)	48.5 (15.3)	52.9 (11.9)	60.8 (10.6)	60.6 (11.2)	0.0002
Sex, *n* (%)						0.0256
Male	66 (55.0)	14 (46.7)	11 (36.7)	20 (66.7)	21 (70.0)	
Female	54 (45.0)	16 (53.3)	19 (63.3)	10 (33.3)	9 (30.0)	
TRT ^1^ (min), mean (SD)	440.2 (95.4)	455.8 (99.3)	462.0 (74.0)	430.5 (116.5)	412.5 (82.4)	0.1570
Mean oxygen saturation, mean (SD)	93.7 (2.0)	95.2 (1.7)	94.1 (1.7)	93.2 (2.1)	92.5 (1.7)	<0.0001
Nadir SPO_2_ ^2^, mean (SD)	81.2 (8.3)	87.5 (6.7)	81.8 (4.5)	80.8 (7.7)	74.6 (8.4)	<0.0001
t89 ^3^ (min), mean (SD)	56.5 (83.2)	37.1 (99.8)	65.0 (79.0)	61.0 (99.3)	63.0 (42.8)	<0.0001
t89 (percent), mean (SD)	13.4 (17.4)	7.1 (17.2)	14.8 (16.0)	16.2 (22.7)	15.4 (10.9)	<0.0001
Average heart rate (bpm ^4^), mean (SD)	65.1 (9.9)	63.1 (9.4)	66.2 (10.7)	64.8 (9.8)	66.5 (9.7)	0.5255
Criteria, *n* (%)						0.1901
3%	36 (30.0)	6 (20.0)	7 (23.3)	13 (43.3)	10 (33.3)	
4%	84 (70.0)	24 (80.0)	23 (76.7)	17 (56.7)	20 (66.7)	

^1^ Total recording time. ^2^ Oxygen saturation. ^3^ Time spent with SpO_2_ < 89%. ^4^ Beats per minute.

**Table 2:** Measures of central tendency for sleep interpretation values by the technicians and software for each diagnostic category.

Variable	Total (*n* = 120)	Snoring (*n* = 30)	Mild (*n* = 30)	Moderate (*n* = 30)	Severe (*n* = 30)	*p*-Value
REI Technician #, median (IQR)	14.7 (25.2)	2.1 (1.9)	7.6 (5.1)	20.1 (7.5)	44.0 (18.0)	<0.0001
REI Polysmith Version 11#, median (IQR), N = 119 ^1^	12.2 (24.5)	2.8 (2.3)	7.0 (4.2)	21.2 (8.7)	42.6 (16.7)	<0.0001
Apnea incidents #, Technician, mean (SD)	63.4 (111.3)	5.0 (6.6)	16.4 (13.3)	51.9 (38.4)	180.5 (170.2)	<0.0001
Apnea incidents #, Software, mean (SD)	83.5 (96.0)	11.9 (13.8)	32.2 (20.9)	87.5 (53.9)	202.2 (108.1)	<0.0001
Hypopnea incidents #, Technician, mean (SD)	70.9 (62.4)	9.7 (7.9)	47.2 (27.2)	93.1 (45.6)	133.5 (64.0)	<0.0001
Hypopnea incidents #, Software, mean (SD)	41.5 (42.9)	6.7 (7.1)	22.7 (13.0)	52.2 (36.2)	84.2 (49.3)	<0.0001

^1^ N = 119 due to the loss of one REI software value for one subject.

**Table 3:** Difference in sleep event interpretation values (technician vs. software variables) by diagnostic category.

Variable	Total (*n* = 120)	Snoring (*n* = 30)	Mild (*n* = 30)	Moderate (*n* = 30)	Severe (*n* = 30)	*p*-Value
REI difference, *n* = 119, median (IQR)	0.5 (4.1)	−0.2 (1.4)	0.75 (4.0)	1.56 (5.6)	1.60 (4.9)	0.0318
Hypopnea incidents difference, median (IQR)	19.0 (45.0)	4.0 (7.0)	23.00 (24.0)	43.0 (40.0)	48.00 (60.0)	<0.0001
Apnea incidents difference, median (IQR)	−16.5 (37.5)	−4.0 (8.0)	−15.0 (14.0)	−39.5 (39.0)	−38.0 (93.0)	<0.0001

**Table 4:** Software diagnoses vs. technician diagnoses of OSA severity. Parenthesis indicates the % of software diagnoses that agreed with technician diagnoses.

	Technician Diagnosis				
Software Diagnosis	Snoring	Mild	Moderate	Severe	Total
Snoring	28 (93.3)	8 (26.7)	0 (0.0)	0 (0.0)	36 (30.0)
Mild	2 (6.7)	22 (73.3)	6 (20.0)	0 (0.0)	30 (25.0)
Moderate	0 (0.0)	0 (0.0)	23 (76.7)	6 (20.0)	29 (24.2)
Severe	0 (0.0)	0 (0.0)	1 (3.3)	24 (80.0)	25 (20.8)
Total	30 (25.0)	30 (25.0)	30 (25.0)	30 (25.0)	120 (100.0)

**Table 5:** Pearson correlation coefficient of REI and Wilcoxon signed rank test; total and OSA technician-determined subgroups.

Variable	r, *p* Value	Wilcoxon Signed Rank Test, *p* Value
ALL N = 119	0.96, <0.0001	0.0134
Mild N = 30	0.45, 0.0129	0.0197
Moderate N = 30	0.56, 0.0012	0.2350
Severe N = 30	0.71, <0.0001	0.0694
Snoring N = 29	0.69, <0.0001	0.1798

**Table 6:** Sensitivity, specificity, PPV, and NPV across OSA diagnostic categories.

	**OSA Diagnostic Categories**				**Moderate/Severe Combined**
	**Snoring**	**Mild**	**Moderate**	**Severe**	**Moderate/Severe**
Sensitivity	93%	73%	77%	80%	90%
Specificity	91%	91%	93%	99%	100%
PPV	78%	73%	79%	96%	100%
NPV	98%	91%	92%	94%	91%

## Data Availability

The data presented in this study are available on request from the corresponding author due to privacy restrictions.
